# Combinatory Effect and Modes of Action of Chrysin and Bone Marrow-Derived Mesenchymal Stem Cells on Streptozotocin/Nicotinamide-Induced Diabetic Rats

**DOI:** 10.3390/ph16010034

**Published:** 2022-12-27

**Authors:** Hesham M. Sayed, Ashraf S. Awaad, Fatma El-Zahraa S. Abdel Rahman, M. Al-Dossari, N. S. Abd El-Gawaad, Osama M. Ahmed

**Affiliations:** 1 Physiology Division, Department of Zoology, Faculty of Science, Beni-Suef University, Beni Suef 62521, Egypt; 2 Department of Anatomy and Embryology, Faculty of Veterinary Medicine, Beni-Suef University, Beni Suef 62521, Egypt; 3 Department of Basic Sciences, Faculty of Oral and Dental Medicine, Nahda University, Beni Suef 62764, Egypt; 4 Department of Physics, Faculty of Science, King Khalid University, Abha 62529, Saudi Arabia

**Keywords:** NA/STZ-induced diabetes mellitus, chrysin, mesenchymal stem cells

## Abstract

The purpose of this study was to see how chrysin and/or bone marrow-derived mesenchymal stem cells (BM-MSCs) affected streptozotocin (STZ)/nicotinamide (NA)-induced diabetic rats as an animal model of type 2 diabetes mellitus (T2DM). Male Wistar rats were given a single intraperitoneal (i.p.) injection of 60 mg STZ/kg bodyweight (bw) 15 min after an i.p. injection of NA (120 mg/kg bw) to induce T2DM. The diabetic rats were given chrysin orally at a dose of 100 mg/kg bw every other day, BM-MSCs intravenously at a dose of 1 × 10^6^ cells/rat/week, and their combination for 30 days after diabetes induction. The rats in the diabetic group displayed impaired oral glucose tolerance and a decrease in liver glycogen content and in serum insulin, C-peptide, and IL-13 levels. They also had significantly upregulated activities in terms of liver glucose-6-phosphatase and glycogen phosphorylase and elevated levels of serum free fatty acids, IL-1β, and TNF-α. In addition, the diabetic rats exhibited a significant elevation in the adipose tissue resistin protein expression level and a significant decrease in the expression of adiponectin, insulin receptor-beta subunit, insulin receptor substrate-1, and insulin receptor substrate-2, which were associated with a decrease in the size of the pancreatic islets and in the number of β-cells and insulin granules in the islets. The treatment of diabetic rats with chrysin and/or BM-MSCs significantly improved the previously deteriorated alterations, with chrysin combined with BM-MSCs being the most effective. Based on these findings, it can be concluded that combining chrysin with BM-MSCs produced greater additive therapeutic value than using them separately in NA/STZ-induced T2DM rats.

## 1. Introduction

Diabetes mellitus (DM) is a debilitating, long-term disease that arises when the body is unable to produce enough insulin or is unable to use the insulin produced efficiently. The primary DM types include type 1, type 2 DM (T2DM), and gestational DM [1]. DM affected approximately 537 million people worldwide in 2021 and is expected to affect 643 million and 784 million people by 2030 and 2045, respectively, according to the International Diabetes Federation (IDF) [2]. Approximately 4.2 million deaths are attributable to DM and its complications [2]. T2DM is marked by a severe elevation in the blood glucose level over the normal values due to both insulin resistance and insufficient insulin secretion [3]. It is the most prevalent type of disease, responsible for around 90% of all cases of diabetes globally [2]. The general aims of diabetes care are to avoid acute decompensation, prevent or postpone complications in the onset of late disease, decrease mortality, and preserve a good quality of life [4].

Medicinal plants and plant-derived natural agents are among the conventional treatments for DM. The therapeutic assessments of constituents from natural sources have attracted several investigators because they have fewer side effects [5,6]. Flavonoids are a broad class of plant-synthesized polyphenolic compounds, and they are secondary metabolites which have a wide variety of pharmacological properties with limited side effects [7,8,9]. Chrysin (5,7-dihydroxyflavone) belongs to the flavone family of polyphenolic compounds, and its major natural origins are various plant extracts, propolis, blue passionflower (*Passiflora caerulea*), and honey [7,10]. Recently, chrysin was reported to demonstrate anti-hyperglycemic, anti-inflammatory, and antioxidant activities in several experimental animals [9,11]. Satyanarayana et al. [12] disclosed that chrysin possessed anti-diabetic, antidyslipidemic, and other properties that reduce inflammation in rats that have developed type 2 diabetes as a result of a high-fat diet. Moreover, chrysin attenuated nicotinamide/streptozotocin (NA/STZ)-induced T2DM rats by improving oral glucose tolerance [13].

Stem cell therapy was successfully implemented in preclinical and clinical DM trials [14,15]. The transplantation of islet cells was formerly thought to be a feasible therapeutic strategy. Due to the scarcity of participants, ethical dilemmas, and the possibility of immunogenicity, this method is not frequently used [16]. One of the most appropriate categories of multipotent adult stem cells, mesenchymal stem cells (MSCs), are readily available from bone marrow (BM), adipose tissues, the umbilical cord, umbilical cord blood, and dental pulp [17,18]. MSCs are undifferentiated cells that possess the properties of self-renewal, the expansion in the presence of culture medium, the activation of endogenous progenitor cells, the modulation of the local environment, the secretion of various factors, and low immunogenicity, but they come with a number of ethical concerns [19,20].

Recent studies have indicated that BM-derived MSCs (BM-MSCs) could potentially exert anti-diabetic effects in a HFD/STZ rat model of T2DM that resulted in the recovery of pancreatic islets, increased insulin secretion, the correction of hyperglycemia, and ameliorated insulin sensitivity [19,20,21,22]. In another animal model, the effect of BM-MSCs therapy in NA/STZ-induced T2DM rats was reported by Hamza et al. [23], who stated their potential effects in ameliorating elevated blood glucose levels.

Several researchers have claimed that the implantation or intravenous injection of MSCs reduced blood glucose levels and improved the regeneration of pancreatic islets [20,21,22,23,24,25]. In T2DM, there is the excessive production of reactive oxygen species and pro-inflammatory and inflammatory cytokines [3,26,27]. Thus, the use of natural products or plant constituents that have both antioxidant and anti-inflammatory properties may potentiate the efficiency of MSCs in the treatment of the disease. The use of a natural flavone, chrysin, which has antioxidant and anti-inflammatory properties [7,10,11,12], may boost stem cell therapy in T2DM. To the best of our knowledge, no such research has yet been published.

Hence, this research sought to examine the therapeutic potential of chrysin and/or BM-MSCs, with a focus on their modes of action, in NA/STZ-induced T2DM in albino rats.

## 2. Materials and Methods

### 2.1. Reagents and Chemicals

DMEM, Dulbecco’s modified Eagle’s high-glucose medium, contains 2 mM L-glutamine without ribonucleosides and ribonucleotides (cat. no. BE12-604F). Penicillin–streptomycin antibiotic mixture (cat. no. 17-602F), 1X phosphate-buffered saline (cat. no. 17-516F), and 0.25% trypsin/1 mM ethylenediaminetetraacetic acid (cat. no. CC-5012) were obtained from Lonza, Belgium. Fetal bovine serum (cat. no. S-001C-BR) was obtained from Life Science Production, Brazil. Sodium hydrogen carbonate (NaHCO_3_) (cat. no. 144-55-8) was delivered from LOBA Chemie (India). Chrysin (5,7-dihydroxyflavone) was purchased from MRM Nutrition, USA. STZ [2-deoxy-2-(3-(methyl-3-nitrosoureido)-Dglucopyranose] (cat. no. 18883-66-4) and NA (cat. no. 98-92-0) were sourced from Sigma-Aldrich (St. Louis, MO, USA).

### 2.2. Experimental Animals

Male rats of the Wistar strain (age: 8–9 weeks, weight: 100–120 g) were sourced from the animal house of the Egyptian Company for the Production of Sera and Vaccines (VACSERA; Helwan, Egypt). To allow acclimation and rule out any concurrent infections, the animals were kept under observation for roughly 15 days prior to the experiment’s start. The chosen animals were kept in well-ventilated polypropylene cages with a regular 12-h light/dark cycle, a humidity of 55 ± 5%, and an ambient temperature of 25 ± 5 °C. The Experimental Animal Ethics Committee for Care and Use of Animals at Beni-Suef University in Egypt gave their clearance (Ethical Approval Number: BSU/FS/2017/21).

### 2.3. Isolation, Culture, and Characterization of BM-MSCs

BM-MSCs were isolated and cultured according to the procedure described by Chaudhary and Rath [28] and in our past publications [29,30,31]. Trypan blue (0.4%) was used to check the vitality of the cultivated cells prior to injection; the results showed that this was 95%. Moreover, reverse transcription polymerase chain reaction (RT-PCR) analysis was used to describe the cells using positive (CD73 and CD105) and negative (CD34 and CD45) BM-MSCS markers [32,33].

### 2.4. Induction of T2DM Animal Model

A single intraperitoneal (i.p.) injection of 60 mg STZ/kg bw was administered after 15 min of NA i.p. injection (120 mg/kg body weight) to the overnight-fasted rats in order to induce T2DM [34,35,36]. STZ was dissolved in citrate buffer (pH 4.5), while NA was dissolved in 0.9% saline solution. After 10 days, STZ-injected rats that had been fasting overnight (10–12 h) were orally given 3 g/kg bw of glucose (dissolved in distilled water) by oral gavage. Blood samples were drawn from the lateral tail vein by puncture after two hours of oral glucose delivery, and blood glucose levels were measured using a glucometer (GM100; Bionime Corporation, Taichung, Taiwan). We selected in our study the rats that had a 2-h blood glucose level more than 200 mg/dL. 

### 2.5. Experimental Protocol

**Figure 1:**
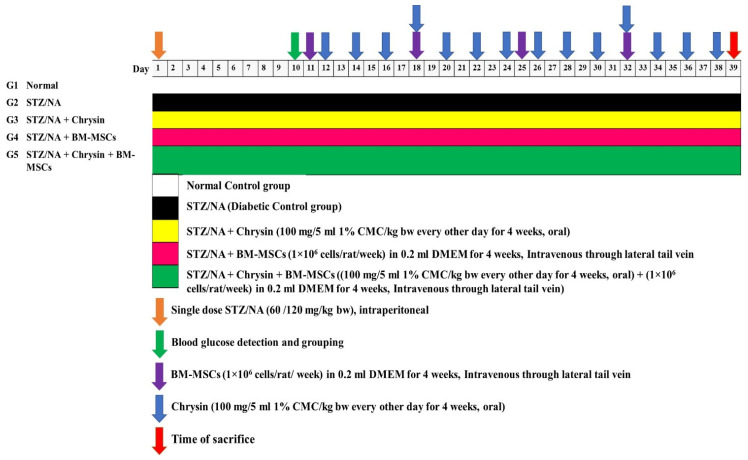
Animal grouping and the experimental procedure.

In this study, a total of 30 male albino rats were randomly assigned to five groups, each consisting of six rats, and were given unlimited access to food and water for the duration of the study, as follows (Figure 1):

Group 1 (normal control): this group were healthy rats and received an equivalent volume of DMEM (0.2 mL/rat) intravenously in the lateral tail vein every week and 1% carboxymethylcellulose (CMC) via oral gavage (5 mL/kg bw) every alternate day.

Group 2 (diabetic control): this group included diabetic rats that received a weekly intravenous injection of DMEM (0.2 mL/rat) in the lateral tail vein and 1% CMC (5 mL/kg bw) via oral gavage every alternate day all the way through the time of study.

Group 3 (diabetic rats treated with chrysin): this group included diabetic rats that orally received chrysin at the dose level of 100 mg/5 mL of 1% CMC/kg bw [12,37] every alternate day until the end of the study (four weeks). Additionally, for a period of four weeks, this group received an equivalent volume of DMEM (0.2 mL/rat) intravenously in the lateral tail vein.

Group 4 (diabetic rats treated with BM-MSCs): this group consisted of six diabetic rats that were treated with 1 × 10^6^ cells/rat in 0.2 mL of DMEM through intravenous injection into lateral tail vein every week for a period of four weeks. Additionally, for four weeks, this group received an oral gavage dosing of 1% CMC (5 mL/kg bw) every other day.

Group 5 (diabetic rats treated with chrysin and BM-MSCs): this group included six diabetic rats, which were treated with BM-MSCs every week in the same way as group 5 and were orally treated with chrysin at a dose level of 100 mg/5 mL of 1% CMC/kg bw every other day for four weeks.

### 2.6. Sampling and Tissue Preparation

At the end of the experiment, the rats were fasted for an entire night before blood samples were taken from the jugular vein under diethyl ether inhalation anaesthesia. The blood samples were drawn into gel and clot activator tubes, allowed to clot, and then centrifuged for 15 min at 3000 rpm. With a Pasteur pipette, the clear, non-hemolyzed supernatant sera were promptly collected into three Eppendorf tubes for each animal and kept at 80 °C until further use for the detection of insulin, C-peptide, tumor necrosis factor-alpha (TNF-α), interleukin-1β (IL-1β), and IL-13 levels. The rats were euthanized, then quickly dissected. Their visceral adipose tissues were removed and kept at 80 °C until resistin, adiponectin, insulin receptor beta subunit (IR-Bs), insulin receptor substrate 1 (IRS-1), and insulin receptor substrate 2 (IRS-2) expression as well as the glucose-6-phosphatase (G-6-Pase), glycogen phosphorylase activities, and glycogen content in the liver were measured. For histological and immunohistochemical analyses, the pancreas was removed and fixed in 10% neutral buffer formalin (NBF).

### 2.7. Oral Glucose Tolerance Test (OGTT)

The OGTT was carried out the day before the sacrifice by measuring the blood glucose levels in overnight-fasted healthy, diabetic control, and normal rats treated with chrysin and/or BM-MSCs at 0, 30, 60, 90, and 120 min after the administration of oral glucose (3 g/kg bw). The blood glucose levels were measured using a BIONIME GM100 glucometer.

### 2.8. Serum Fasting C-Peptide and Insulin Analysis

Following the manufacturer’s instructions, a sandwich enzyme-linked immunosorbent assay (ELISA) kit from MyBioSource (USA) was used to assess the levels of fasting serum insulin and C-peptide (Catalog Number: MBS724709 and MBS704133, respectively).

### 2.9. Serum-Free Fatty Acids (FFAs)

The method outlined by Duncombe [38] was used to estimate the serum FFAs level.

### 2.10. Detection of Liver Glycogen Content, G-6-Pase, and Glycogen Phosphorylase Activities

Using reagents prepared in the lab, the liver glycogen content was determined according to the method of Seifter et al. [39], and G-6-Pase and glycogen phosphorylase activities were detected based on the procedures of Begum et al. [40] and Stalmans and Hers [41], respectively.

### 2.11. Detection of Serum IL-1β, TNF-α, and IL-13

According to the manufacturer’s instructions, the RayBio^®^ Rat IL-1 ELISA kit (Catalog No: ELR-IL1) and the BioLegend Rat TNF-ELISA kit (Catalog No: 438205) procured from RayBiotech (Norcross, GA, USA) and BioLegend, Inc (San Diego, CA, USA), respectively, were used to measure the blood levels of IL-1 and TNF- as pro-inflammatory cytokines. Using the rat IL-13 ELISA kit (Catalog No: MBS175932) acquired from MyBioSource (San Diego, CA, USA) and used in accordance with MyBioSource’s instructions, serum IL-13 was identified as an anti-inflammatory cytokine.

### 2.12. Detection of BM-MSCs’ Different CDs’ mRNA Expression

The total RNA was extracted from the adipose tissues using the Direct-zol RNA Miniprep kit (Zymo Research, Irvine, CA, USA) (Catalog No. R2050) based on the manufacturer’s instructions. Thermo Scientific’s RevertAid First-Strand cDNA synthesis kit (Catalog No. AB1454LDB) was used to create cloned DNA (cDNA), which was then amplified using the Thermal cycler Techne 312 in the presence of specific forward and reverse primers (Fisher Scientific, Leicestershire, LE11 5RG). RNA was reverse transcribed into cDNA in a single step, and the cDNA that was created was amplified using a Techne 312 Thermocycler. The Cleaver Scientific Ltd. MP-300V Power System electrophoresed PCR products at 90 volts on a 1.5% agarose gel stained with ethidium bromide in a 1X Tris borate EDTA buffer (TBE; pH 8.3–8.5). A UV transilluminator was used to observe the electrophoretic image, which was then captured on camera and analyzed using the ImageJ 1.52v java 1.8.0 112 (64-bit) application [42]. The primer pair sequences used included: for CD73 5’-TGCATCGATATGGCCAGTCC-3’ (forward) and 5’-AATCCATCCCCACCGTTGAC-3’ (reverse) [32], for CD105 5’-ACTGAGTTGCACATCTGGGG-3’ (forward) and 5’-TTCCGAAGTGGTGGTAAGCC-3’ (reverse) [32], for CD34 5’-AGCCATGTGCTCACACATCA-3’ (forward) and 5’-CAAACACTCGGGCCTAACCT-3’ (reverse) [32], and for CD45 5’-TTGCTCCCCATCCGATAAGAC-3’ (forward) and 5’-AGCGTGGATGAAAAACCATCG-3’ (reverse) [32]. The primers were obtained from LIGO Scientific Collaboration through National BioLab, Cairo, Egypt.

### 2.13. Western Blotting Analysis

The adipose tissue sample was homogenised using the ReadyPrepTM protein extraction kit (Bio-Rad Inc; Catalog No: 163-2086). The Bradford protein assay kit (SK3041) for quantitative protein analysis offered by Bio Basic Inc. was used in accordance with the manufacturer’s instructions (Markham, Ontario L3R 8T4 Canada). In an equal volume of 2X Laemmli sample buffer that also contained SDS (4%), 2-mercaptoehtanol (10%), glycerol (20%), bromophenol blue (0.004%), and Tris HCl (0.125 M), 20 μg of the protein concentration of each sample was loaded. The pH was measured and set at 6.8. Each of the previous mixtures was boiled at 95 °C for 5 min to induce protein denaturation prior to loading on polyacrylamide gel electrophoresis. The polyacrylamide gels were created using Bio-Rad Laboratories Inc.’s TGX Stain-FreeTM FastCastTM Acrylamide Kit (SDS-PAGE) (Catalog No. 161-0181). According to the manufacturer’s instructions, the SDS-PAGE TGX Stain-Free FastCast was created. From below to above, the gel was built in a transfer sandwich (filter paper, PVDF membrane, and gel and filter paper). The sandwich was placed in a transfer tank containing 1X transfer buffer (Tris (25 mM), glycine (190 mM), and methanol (20%).

The blot was passed through the Bio-Rad Trans-Blot Turbo for 7 min at 25 V to allow the protein bands to move from the gel to the membrane. The membrane was blocked in Tris-buffered saline with Tween 20 (TBST) buffer and 3% bovine serum albumin for 1 h at room temperature. Adiponectin, resistin, IR-Bs, IRS-1, and IRS-2 primary antibodies were purchased. Following the manufacturer’s directions, these primary antibodies were diluted in TBST. The blotted target protein was incubated with each primary antibody solution for the entire night at 4 °C. For five minutes, the blot was rinsed three–five times with TBST. The secondary antibody solution (goat anti-rabbit IgG-HRP-1 mg Goat mab-Novus Biologicals) was HRP-conjugated and treated with the blotted target protein for 1 h at room temperature. With the TBST buffer, the blot was washed three–five times for 5 min. Clarity TM Western ECL Substrate Bio-Rad (Catalog No: 170-5060), a chemiluminescent substrate, was added to the blot in accordance with the manufacturer’s instructions. Briefly, solution A (Clarity Western luminal/enhancer solution) and solution B were blended in equal parts (peroxidase solution). The chemiluminescent signals were gathered using a CCD camera-based imager. Using protein normalization on the ChemiDoc MP imager, image analysis software was used to compare the band intensity of the target proteins to that of the housekeeping protein β-actin in the control sample.

### 2.14. Histological Investigation

Each rat’s pancreas was removed following dissection after four weeks, and it was then fixed in 10% (*v*/*v*) NBF for 48 h. All specimens were then cleared, paraffin-embedded for 24 h at 56 °C in a hot air oven, and transferred to 70% alcohol for histological analysis. The paraffin wax blocks for the pancreas were ready for cutting at 5 m. Trichrome-PAS was used to stain the acquired tissue sections [43,44]. A high-power microscope was used for the investigation, and a camera was used to create photomicrographs.

### 2.15. Immunohistochemical Investigation

Each pancreas was routinely treated for paraffin embedding after being fixed in 10% (*v*/*v*) NBF. Sections of the tissue blocks that were 5-µm thick were taken off. Rat insulin (polyclonal antibody) immunohistochemistry was carried out using the streptavidin-biotin-peroxidase staining technique [45]. All sections were incubated with the same amount of antibodies at the same time and under the same conditions, allowing the results of the immunostaining to be compared between the various experimental groups. Using a photomicrograph camera and high-power light microscopy, the binding of antibodies was studied. Three random fields were estimated for each section. The number of sections in each group was six. ImageJ (1.51 d), a free software program, was used to measure the integrated density and area fraction of immune positivity of insulin granules [42]. 

### 2.16. Statistical Analysis

The mean and standard error served as a general illustration of the results. The GraphPad Prism software (version 8.0.2; GraphPad Software, San Diego, CA, USA) was used to perform a one-way analysis of variance (ANOVA), followed by a Tukey’s post hoc analysis test to assess the statistical differences between the groups. Significant differences were defined as those with a *p*-value < 0.05. Percentage changes were calculated by comparing the diabetic control with normal and diabetic treated groups with diabetic control by using the equation: % change = [(Final–Initial)/Initial] × 100. The area under curve (AUC) for the OGTT was also calculated the GraphPad Prism software. 

## 3. Results

### 3.1. Characterization of MSCs

**Figure 2:**
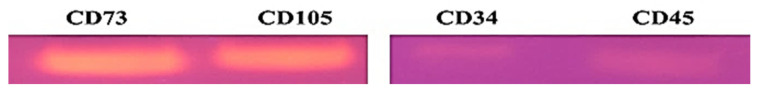
RT-PCR analysis showing mRNA expression of CD73, CD105, CD34, and CD45 in rat MSCs. CD73 and CD105 were strongly expressed from MSCs. However, CD34 and CD45 were weakly expressed.

Under inverted microscopy, the separated MSCS were verified for adherence and spindle shape, after which MSC-specific markers including CD73 and CD105 were expressed, with CD34 and CD105 being negatively expressed (Figure 2).

### 3.2. Effect of Chrysin and BM-MSCs on Oral Glucose Tolerance in NA/STZ-Induced Diabetic Rats

**Figure 3:**
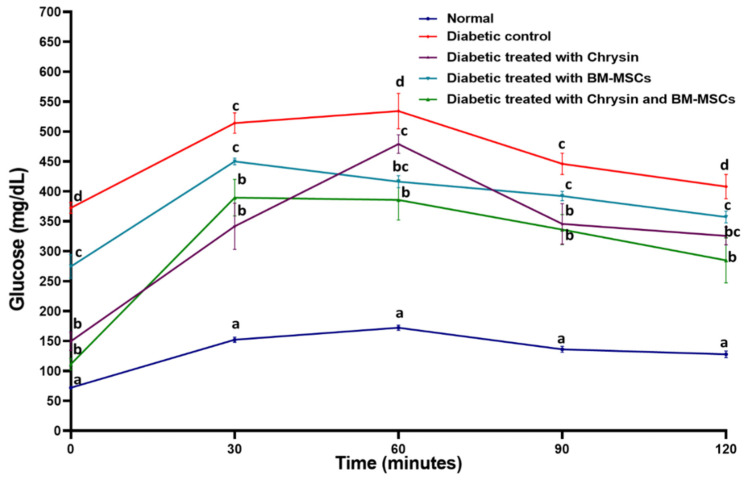
OGTT of normal, diabetic control, and diabetic rats treated with chrysin and BM-MSCs. Data are expressed as mean ± SE (n = 6). At *p* < 0.05, the means (a, b, c, and d) are significantly different from one another.

Following the delivery of glucose at a dose of 3 g/kg bw, blood glucose levels in rats were assessed at 0, 30, 60, 90, and 120 min (Figure 3). The blood glucose levels of the diabetic control rats continued to rise up until 60 min and they subsequently decreased at 90 and 120 min. The blood glucose levels were considerably (*p* < 0.05) elevated at all phases of the OGTT following the administration of glucose relative to normal rats. A significant (*p* < 0.05) improvement in the increased glucose levels was seen in the diabetic rats treated with chrysin, BM-MSCs, and their combination. The combination was discovered to be the most effective method for lowering the blood glucose levels of diabetic rats when they were fasting, and the effectiveness peaked 60, 90, and 120 min after oral glucose administration. 

The calculated AUC for the OGTT in Table 1 was significantly (*p* < 0.05) increased in the diabetic rats when compared to the normal rats. The treatment of diabetic rats with chrysin, BM-MSCs, and their combination produced a significant decrease (*p* < 0.05) in the AUC. The recorded percentage changes were −30.09, −19.43, and −36.03, respectively. Thus, the combinatory effect was the most potent in improving the oral glucose tolerance. 

### 3.3. Effect of Chrysin and BM-MSCs on the Serum Insulin and C-Peptide Levels in NA/STZ-Induced Diabetic Rats

When compared to the normal control rats, the diabetic rats’ serum levels of insulin and C-peptide significantly (*p* < 0.05) reduced, with percentage changes of 64.86 and 71.64%, respectively. The serum insulin levels in diabetic rats treated with chrysin, BM-MSCs, and chrysin plus BM-MSCs increased significantly (*p* < 0.05), with percentage changes of 122.98, 136.90, and 138.56%, respectively, when compared with the diabetic control. Additionally, there were changes in the serum C-peptide level of 101.34, 143.70, and 130.28%, respectively (Table 2). Although the serum insulin and C-peptide levels significantly (*p* < 0.05) increased in the diabetic rats treated with chrysin and/or BM-MSCs, they did not return to normal levels. They remained significantly lower (*p* < 0.05) than the normal values. 

### 3.4. Effect of Chrysin and BM-MSCs on Serum Free Fatty Acid Levels in NA/STZ-Induced Diabetic Rats

Table 3 demonstrates the effect of chrysin and BM-MSCs on the FFAs of diabetic rats. When compared to the control group, the NA/STZ-induced diabetic rats showed a significant increase (*p* < 0.05) in serum FFAs. The increased serum FFAs levels were significantly reduced (*p* < 0.05) in diabetic rats after treatment with chrysin and BM-MSCs. The serum FFAs levels of diabetic groups treated with chrysin and BM-MSCs returned to values that were not significantly (*p* > 0.05) different to normal levels.

### 3.5. Effects on Liver Glycogen Content, G-6-Pase, and Glycogen Phosphorylase Activities in NA/STZ-Induced Diabetic Rats

In comparison with the normal group, the diabetic control rats’ liver glycogen content showed a significant decrease (*p* < 0.05); the measured percentage drop was −52.69%. The liver glycogen content of NA/STZ-induced diabetic rats significantly increased (*p* < 0.05) after receiving chrysin, BM-MSCs, or chrysin plus BM-MSCs. The liver glycogen content of NA/STZ-induced diabetic rats increased most effectively with chrysin plus BM-MSCs; the measured percentage increase in comparison to the diabetic control was 68.20%. Although the liver glycogen content significantly (*p* < 0.05) increased in the diabetic rats treated with chrysin and/or BM-MSCs, it did not return to normal. It remained significantly lower (*p* < 0.05) than the normal values (Table 4). 

On the other hand, compared to the normal group, the NA/STZ-induced diabetic rats’ liver G-6-Pase and glycogen phosphorylase activities showed a substantial increase (*p* < 0.05); the observed percentage changes were 93.08% and 120.53%, respectively. The enhanced enzyme activities in NA/STZ-induced diabetic rats were significantly improved (*p* < 0.05) by the administration of chrysin, BM-MSCs, and chrysin combined with BM-MSCs; however, they remained significantly higher than normal levels (*p* < 0.05) (Table 4). 

### 3.6. Effect on IL-1β, TNF-α, and IL-13 Levels

When compared to the serum of the normal rats, the levels of the pro-inflammatory cytokines IL-1 and TNF-α in the NA/STZ-induced diabetic rats showed a substantial (*p* < 0.05) elevation. The serum levels of IL-1 and TNF-α were significantly (*p* < 0.05) reduced in the diabetic rats treated with chrysin, BM-MSCs, and chrysin + BM-MSCs. When compared to the serum of normal rats, the anti-inflammatory cytokine IL-13, however, showed a substantial (*p* < 0.05) drop in diabetic rats. Chrysin, BM-MSCs, and chrysin plus BM-MSCs treatment resulted in a significant (*p* < 0.05) rise in serum IL-13 levels in diabetic rats (Table 5). Chrysin plus BM-MSCs treatment was the most potent treatment in decreasing the elevated serum TNF-α level in diabetic rats compared to the normal level. 

### 3.7. Effects on the Protein Expression Levels of Resistin and Adiponectin

**Figure 4:**
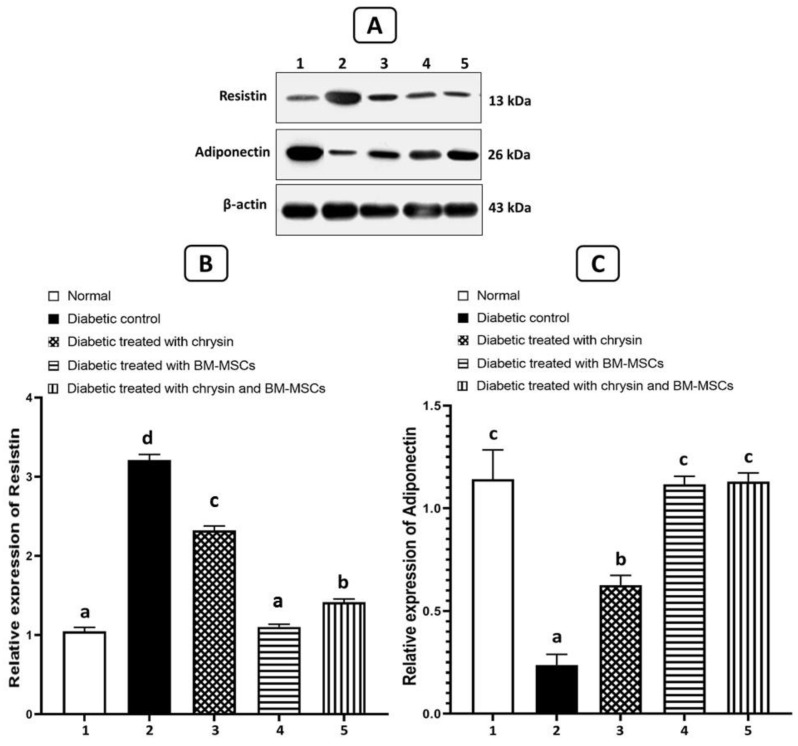
Effects of chrysin and BM-MSCs on resistin (**B**) and adiponectin (**C**) in adipose tissue in NA/STZ-induced diabetic rats. Western blot analysis was used to measure the expressions of resistin and adiponectin in the adipose tissues of the experimental groups. Representative immunoblots for the quantification of resistin and adiponectin proteins are depicted in (**A**). Data are expressed as mean ± SE (n = 3). Means (with various symbols: a, b, c, and d) for each parameter differ significantly at *p* < 0.05.

Figure 4 shows the data on how resistin and adiponectin expressions in the adipose tissues of NA/STZ-induced T2DM rats were affected by chrysin, BM-MSCs, and chrysin plus MSCs. When compared to normal rats, NA/STZ-induced diabetic rats showed a significantly higher level of resistin expression (*p* < 0.05) and a significantly lower level of adiponectin expression relative to β-actin. As opposed to the diabetic control group, diabetic rats treated with chrysin, BM-MSCs, and chrysin plus MSCs showed a significant enhancement in every parameter (*p* < 0.05). 

### 3.8. Effects on the Protein Expression Levels of IR-Βs, IRS-1, and IRS-2

**Figure 5:**
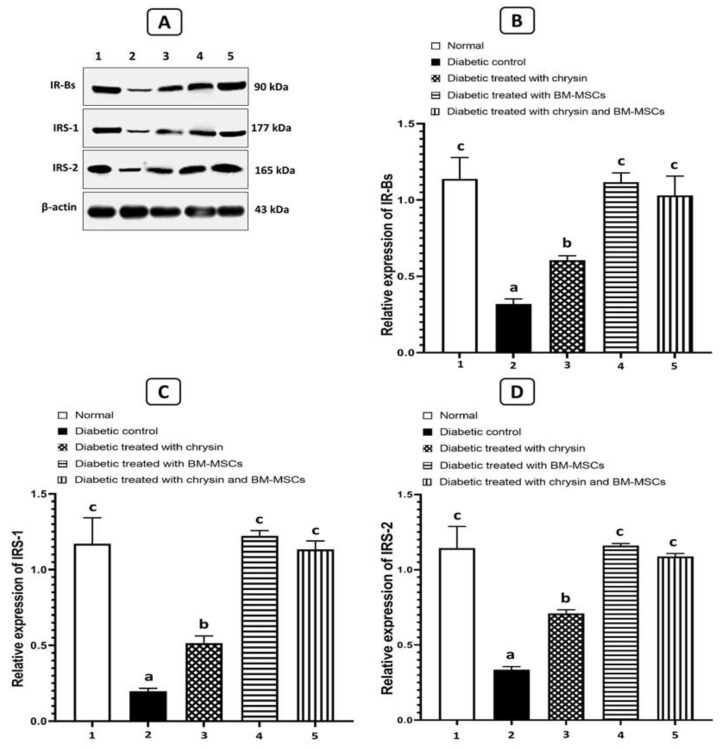
Effects of chrysin and BM-MSCs on IR-Βs (**B**), IRS-1 (**C**), and IRS-2 (**D**) in adipose tissue in NA/STZ-induced diabetic rats. Western blot analysis was used to measure the expressions of IR-Βs, IRS-1, and IRS-2 in the adipose tissues of the experimental groups. Representative immunoblots for quantification of resistin and adiponectin proteins are depicted in (**A**). Data are expressed as mean ± SE (n = 3). Means (with various symbols: a, b, and c) for each parameter differ significantly at *p* < 0.05.

Figure 5 shows the data concerning the effects of chrysin, BM-MSCs, and chrysin plus MSCs on IR-Bs, IRS-1, and IRS-2 expressions relative to the expression of β-actin in the adipose tissues of NA/STZ-induced T2DM rats. When compared to the expression of β-actin in the normal rats, the diabetic rats showed a significantly lower level of IR-s, IRS-1, and IRS-2 expression (*p* < 0.05). In contrast, diabetic rats given chrysin, BM-MSCs, or chrysin plus MSCs showed a significant improvement in all parameters (*p* < 0.05) when compared to the diabetic control.

### 3.9. Effects of Chrysin and BM-MSCs on Histological Changes in the Pancreas in NA/STZ-Induced Diabetic Rats

**Figure 6:**
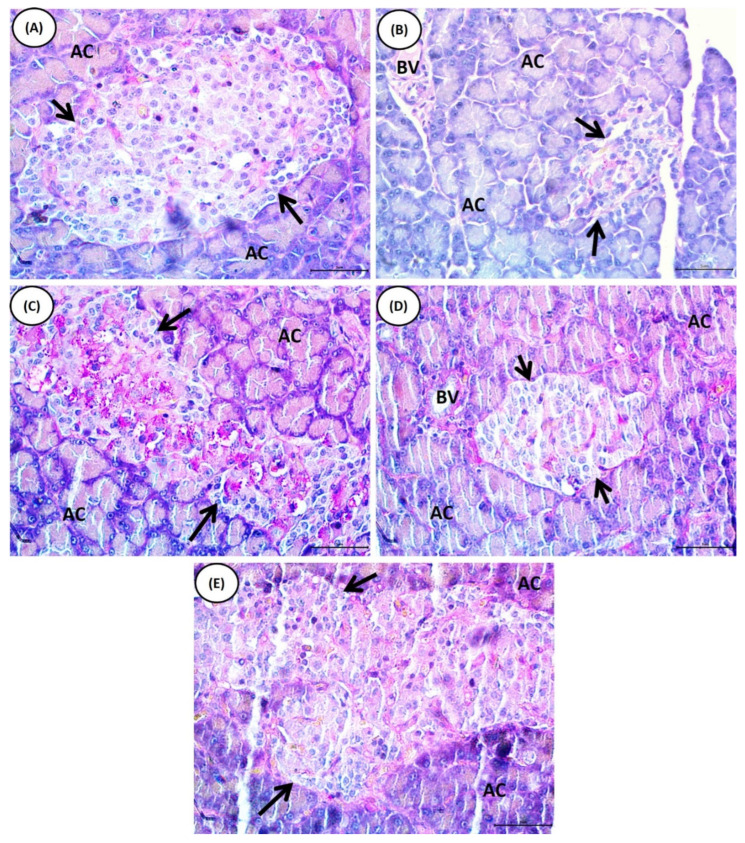
Trichrome-PASS-stained rat pancreas photomicrographs of the normal, diabetic control, and treated groups. (**A**): Pancreas sections of normal rats showing normal histological architecture formed of closely packed lobules of pancreatic acinar cells and islets of Langerhans embedded in the exocrine portions. (**B**): Pancreas sections of NA/STZ-administrated rats showing a marked decrease in the size and number of β-cells and islets. Pancreas sections of NA/STZ-administrated rats treated with chrysin (**C**), BM-MSCs (**D**) and chrysin plus BM-MSCs (**E**) showing marked improvement regarding pancreas histological changes, resulting in a nearly normal structure in terms of the islets of Langerhans. (Arrows: islets of Langerhans; AC: acinar cells; BV: blood vessel).

Figure 6 shows the pancreatic histological architectures of normal, NA/STZ-administered, and NA/STZ-administered rats treated with chrysin, BM-MSCs, and their combination. The normal control group’s pancreas sections demonstrated a typical histological architecture composed of densely packed lobules of pancreatic acinar cells. The alpha cells and exocrine sections contained the islets of Langerhans (Figure 6A). The endocrine sections of the pancreas of NA/STZ-administered rats showed histological abnormalities, which were manifested by a notable reduction in the size and number of cells and islets in other portions (Figure 6B). The NA/STZ-administered group was treated with chrysin (Figure 6C), BM-MSCs (Figure 6D), and their combination (Figure 6E) and exhibited a marked improvement in pancreas histological abnormalities, resulting in virtually normal structures in terms of the islets of Langerhans.

### 3.10. Effects of Chrysin and BM-MSCs on Pancreas Immunohistochemical Staining of Insulin in NA/STZ-Induced Diabetic Rats

**Figure 7:**
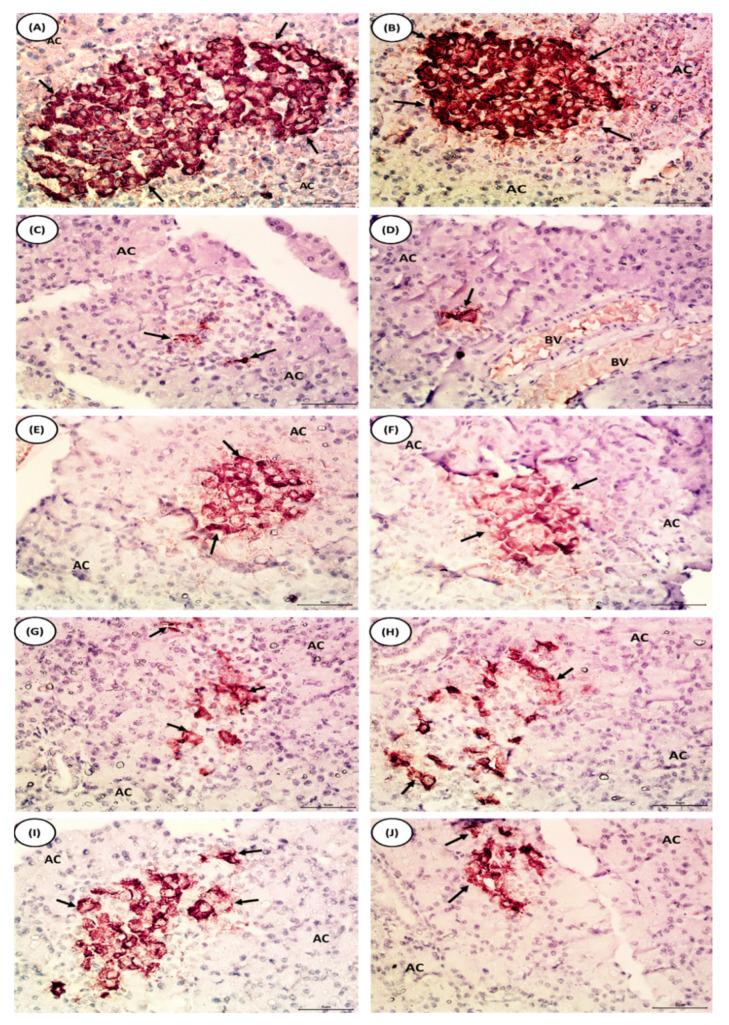
Photomicrographs of immunohistochemical staining of insulin in pancreatic islets of the normal, diabetic control, and diabetic treated rats. (**A**,**B**) Pancreas section of rats from normal group showing strong immunoreactivity of insulin in β-cells which are distributed over the pancreatic islets (arrows) and stained with deep brown color. (**C**,**D**) Pancreas section of rats from NA/STZ-induced diabetic group showing evident decline in the immunohistochemical expression of insulin in islet of Langerhans (arrows). (**E**,**F**) In the pancreas of diabetic rats treated with chrysin, the apparent marked increase in number and area of β-cells with dense immunohistochemical staining of insulin granules is evident in comparison with NA/STZ-induced diabetic group (arrows). (**G**,**H**) Pancreas section of rats treated with BM-MSCs showing an evident increase in insulin expressing β-cells (arrows). (**I**,**J**) In the pancreas of diabetic group rats treated with chrysin plus BM-MSCs, the apparent marked increase in number and area of β-cells is evident in comparison with NA/STZ-induced diabetic group (arrows).

Photomicrographs of the immunohistochemical staining of insulin pancreatic islets of all groups were analyzed by using the ImageJ 1.52v java 1.8.0_112 (64-bit) program [42] to the achieve integrated density and area fraction. The normal rats displayed an intense insulin immunopositive reaction to anti-insulin antibodies, as brown granules occupied most of the islets (Figure 6A,B). The immunoreactivity for anti-insulin antibodies in the rat pancreas in the NA/STZ-induced diabetic group was significantly decreased (*p* < 0.05) in terms of the integrated density and area fraction (Figure 7C,D). Treatment with chrysin (Figure 7E,F), BM-MSCs (Figure 7G,H), and chrysin plus BM-MSCs (Figure 7I,J) resulted in a significant increase (*p* < 0.05) in the integrated density and area fraction when compared to the NA/STZ-induced diabetic group (Table 6).

## 4. Discussion 

Increased levels of blood glucose, insulin resistance, and relative insulin deficiency are the characteristics of T2DM [11,35]. Thus, the physiological modulation of glucose levels in the blood can be restored either by increasing the β-cell mass or by enhancing the tissue insulin sensitivity. Similar to endothelial progenitor cells, BM-MSCs can help β-cells recover from injury [24,46] owing to their higher differentiation potential, immunosuppressive characteristics, anti-inflammatory activities, [46] and supportive micro-environmental niche [47]. Various medicinal plants have been estimated to contain flavonoids that exert beneficial and safe effects on diabetes relative to conventionally synthetic drugs [7,48,49]. The NA/STZ rat model was applied as a T2DM model based on NA’s protective effects against STZ’s toxic effects on β-cells [50]. The severity of DM is, however, somewhat lessened if NA is administered prior to STZ, resulting in a state that is similar to T2DM, with impaired insulin sensitivity [51,52,53]. As a result, the NA/STZ-induced diabetic rat model was chosen for our investigation to examine the effects of chrysin, BM-MSCs, and chrysin plus BM-MSCs.

In the present investigation, rats that had been given NA/STZ to induce T2DM showed impaired oral glucose tolerance, a significant increase in the AUC of the OGTT, and a significant reduction in serum levels of C-peptide and insulin. These outcomes line up with earlier published reports [35,42,54].

The chrysin therapy, in the present study, resulted in a significant improvement in the deteriorated oral glucose tolerance and significant decrease in the AUC of the OGTT combined with a large increase in the fallen serum insulin level in the NA/STZ-induced diabetic rats. These findings corroborate those of Satyanarayana et al. [12], who reported that the oral administration of chrysin for 10 consecutive days improved the OGTT and raised the serum insulin level after 45 days of treatment in diabetic rats. Additionally, our current research backs up the findings of Anitha and Rajadurai [13], who claimed that the prevention of defective insulin-signaling molecules and glucose tolerance was responsible for the anti-diabetic activity of chrysin after 30-days post-induction of T2DM in rats. Similar to this, Ramrez-Espinosa et al. [11] observed that the insulin-sensitizing activity of chrysin caused a significant decrease in serum glucose levels 10 days after oral chrysin administration to NA/STZ-induced diabetic mice.

**Figure 8:**
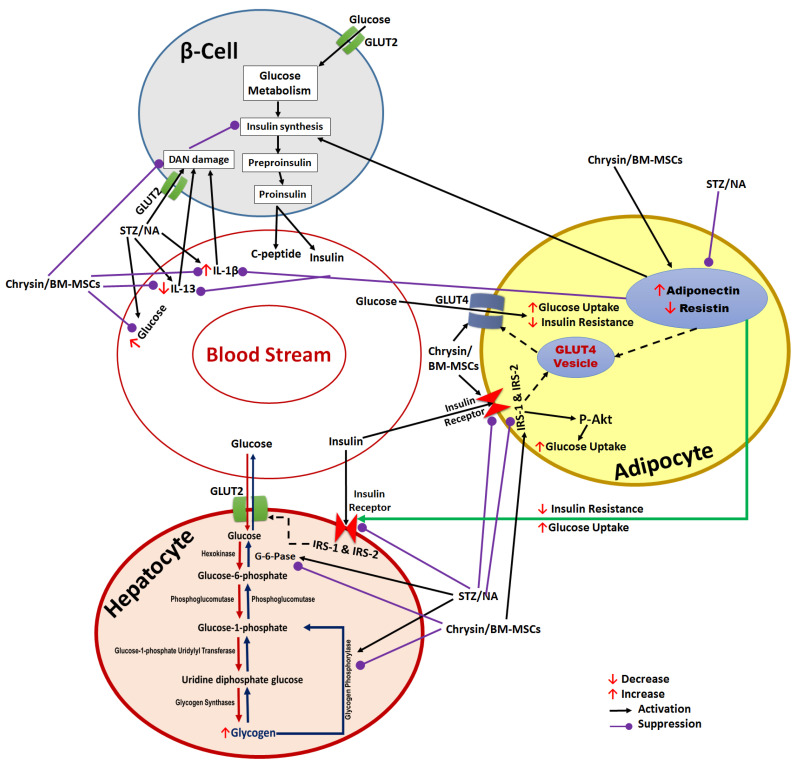
Diagrammatic representation showing the target effects of BM-MSCs and chrysin in NA/STZ-induced type 2 diabetes, illustrating the immunomodulatory and anti-diabetic effects. BM-MSCs: bone marrow-derived mesenchymal stem cells; TNF-α: tumor necrosis factor-alpha; IL-1β; interleukin-1β; IL-13; interleukin-13; NA: nicotinamide; STZ; streptozotocin; Glut4: glucose transporter 4; Glut2: glucose transporter 2; IRS-1: insulin receptor substrate-1; IRS-2: insulin receptor substrate-2; IR-Βs: insulin receptor-beta subunit.

Other researchers have also published their analyses of chrysin’s anti-diabetic benefits [7,10,55]. The current investigation showed that diabetic rats treated with chrysin saw a rise in the C-peptide level that was consistent with the pattern of changes in the serum insulin level. The blood C-peptide level was utilized as a good predictor of any change in insulin levels since it was co-secreted with insulin from the pancreatic cells as a consequence of the conversion of proinsulin to insulin (Figure 8) [56]. In terms of other models of T2DM, our results support those of Li et al. [19], Hussien et al. [20], Si et al. [22], and Hao et al. [57], who reported that BM-MSCs infusions enhanced the status of hyperglycemia, serum C-peptide, and insulin in T2D rats induced by a fat-fed diet and STZ. The ameliorative effect of chrysin and BM-MSCs was also confirmed by the improvement in the histological architecture of the Trichrome-PASS stain and the increase in insulin immunoreactivity. The pancreas of control animals exhibited the typical lobular histological architecture of the islets of Langerhans and pancreatic acini. In the present study, the islet numbers and diameter were markedly reduced in NA/STZ-induced diabetic rats, supporting the results of Al-Hariri et al. [58], who observed fewer Langerhans islets in diabetic rats than in control rats. The diabetic rats treated with chrysin, BM-MSCs, and chrysin plus BM-MSCs indicated a marked improvement in the amount and size of Langerhans islets. These outcomes matched those of Hamza et al. [23], who reported that the treatment of NA/STZ-induced diabetic rats with BM-MSCs restored the normal histology of pancreatic islets. Moreover, the immune reactivity for anti-insulin antibodies was decreased markedly in diabetic rats. The treatment with chrysin, BM-MSCs, and their combination resulted in a high immunoreactivity to anti-insulin antibodies, which was demonstrated by the increased integrated density and area fraction. The enhanced histological architecture and integrity of the pancreatic islet can be attributed to the capacity of BM-MSCs to facilitate the regeneration of endogenous pancreatic islet β-cells by migrating to the damaged islet cells and their involvement in the repair processes by secreting different growth factors and cytokines in both paracrine and autocrine operations. In addition, chrysin acts as an insulin sensitizer and exerts anti-inflammatory effects [59].

According to reports, elevated FFA levels in the blood of NA/STZ-injected rats may lead to insulin resistance and decreased glucose tolerance [60,61]. According to Oh et al. [62], chronically high FFA levels resulted in insulin resistance and β-cell malfunction. As a result, reducing high plasma FFA levels may be a key therapeutic objective in T2DM and obesity. In the present investigation, treatments with chrysin and BM-MSCs effectively reduced the high levels of serum FFAs in the NA/STZ-induced diabetic rats. FFAs affect the expression of genes, especially those involved in the lipid and glucose metabolisms.

Through a variety of mechanisms, including lipotoxicity [63] caused by the toxin effects on pancreatic β-cells, insulin-stimulated glucose transport inhibition [64], and increased adipose tissue lipolysis, elevated levels of FFAs reduce insulin sensitivity. Subsequent FFAs flow to non-adipose tissue, causing excessive endogenous glucose generation, insulin resistance, and the progression of T2DM [65]. Thus, in diabetic rats treated with chrysin and BM-MSCs, the decrease in serum FFAs enhanced the secretory response of β-cells and heightened their insulin-sensitizing properties. 

Adiponectin, IR-Bs, IRS-1, and IRS-2 levels were significantly reduced in NA/STZ-induced T2DM rats, while resistin levels were significantly greater. This is an important finding regarding the current study. These findings are corroborated by those of Abdel Aziz et al. [35], who found that there was a large rise in resistin in NA/STZ-induced diabetic rats compared to normal rats, while the mRNA expression of adiponectin and IRs of adipose tissues demonstrated were significantly decreased. Chrysin and BM-MSCs increased the expression of adiponectin, IR-Bs, IRS-1, and IRS-2 in adipose tissue, which can be attributed to a reduction in the levels of serum FFAs in NA/STZ-induced diabetic rats. Adiponectin increases the oxidation of a muscle’s fatty acid and glucose transport via the phosphorylation of AMP-activated protein kinase (AMPK) or Akt [66] and Acetyl-CoA carboxylase inhibition [67], suppresses gluconeogenesis in the liver as a result of lowering G-6-Pase and phosphoenolpyruvate carboxylase expression [68], and increases energy consumption and fatty acid combustion, leading to low amounts of triglyceride in the skeletal muscles and the liver [69]. Through its modulation of glucose and lipid transcriptional factor expression, adiponectin may act as a direct regulator of glucose utilization in adipocytes and adipose tissues [68,70]. The well-known negative relationship between plasma adiponectin levels and insulin resistance suggests that adiponectin is the master regulator of insulin sensitivity and glucose homeostasis [71,72], possibly by activating AMPK. According to previous studies and our results, chrysin and BM-MSCs’ ability to significantly increase adiponectin expression may be the cause of their anti-diabetic impact in NA/STZ-induced diabetic rats. Chrysin and BM-MSC treatment of diabetic rats increased the expression of the IR-Bs, IRS-1, and IRS-2 genes in a manner similar to that of adiponectin in adipose tissues. The increased expression of the IR-Bs, IRS-1, IRS-2, and phosphatidylinositol 3-kinase (PI3K) proteins may play a role in the mechanism underlying insulin signaling.

The results of the current investigation revealed that the expression of resistin in adipose tissues was significantly higher than it was in normal rats, which was consistent with the reduced glucose tolerance in diabetic rats. The findings of Kim et al. [73], who revealed that resistin is produced only in adipocytes and is associated with aspects relating to insulin resistance and obesity, confirm these findings. Additionally, Rajala et al. [74] observed that in DIO mice and Lep ob/ob mice, circulatory resistin concentrations were dramatically elevated and strongly correlated with the increasing levels of glucose, insulin, and lipids. Resistin increases endogenous glucose synthesis, as shown by decreased glycogen synthase activity and the enhanced insulin-independent expression of the genes controlling the hepatic gluconeogenic enzymes G-6-Pase and phosphoenolpyruvate carboxy kinase in liver cells [75,76]. When given to diabetic rats, chrysin and BM-MSCs drastically decreased the expression of resistin in the adipose tissues. The significant anti-diabetic impact of chrysin and BM-MSCs in our investigation could therefore be attributable, at least in part, to their resistin-modulating activity (Figure 8).

The homeostasis of glucose depends on an appropriate balance in terms of glycolysis, glycogen metabolism, and gluconeogenesis [77,78]. Liver glycogen can be considered a good marker for evaluating the hypoglycemic effect of any medicine, particularly in research animals [79,80]. In the current investigation, rats that were administered NA/STZ to cause diabetes showed a decrease in the amount of hepatic glycogen in addition to a marked rise in the activities of the enzymes G-6-Pase and glycogen phosphorylase in comparison to those of control rats. These findings agree with those of Ali et al. [80], Ahmed et al. [81], Pari and Suman [82], Morral [83], and Ahmed [84], who revealed that NA/STZ-induced diabetic rats had decreased hepatic glycogen content and higher hepatic G-6-Pase and glycogen phosphorylase activity. Our research study results expressed a considerable increase in the hepatic glycogen and a significant reduction in the activity of hepatic G-6-Pase and glycogen phosphorylase, possibly due to the restoration of the insulin level that encourages glycogenesis and the increased glucose uptake, after the administration of chrysin, BM-MSCs, and chrysin plus BM-MSCs for treatment, resulting in decreased glucose production and hence an improvement in blood glucose (Figure 8).

By triggering numerous inflammatory responses, pro-inflammatory mediators play a significant role in the emergence of IR and T2DM. The β-cells of pancreatic islets and insulin-sensitive tissues, particularly adipose tissue, experience metabolic stress as a result of elevated blood glucose levels. Numerous pro-inflammatory cytokines, particularly the master pro-inflammatory mediator IL-1β, TNF-α, IL-1, IL-6, and IL-10, are released from these tissues and cause local and systemic chronic low-grade inflammation [85,86]. The activation of pro-inflammatory mediators causes β-cells to undergo apoptosis, amyloidosis, and fibrosis. Once inflammation has occurred, it triggers its harmful effects on the pancreatic islet β-cells by reducing their ability to secrete insulin. Similar to this, IL-β contributes to the progression of insulin resistance in the peripheral tissues by causing inflammation in those tissues as a result of those tissues’ diminished capacity to use insulin in response to glucose [85]. In STZ-NA-induced diabetic mice, alterations in blood glucose levels and insulin levels are accompanied by elevated levels of IL-1β and TNF-α, which is consistent with our findings and has been shown to accompany pancreatic β-cell dysfunction [86,87]. The current investigation showed that chrysin, BM-MSCs, and chrysin plus BM-MSCs therapy of NA/STZ-induced T2DM rats dramatically reduced serum levels and the production of increased pro-inflammatory cytokines IL-1β and TNF-α. These findings concur with those of Ahad et al. [88], who found that the TNF-α pathway was inhibited by chrysin for 16 weeks, resulting in a decrease in the release of pro-inflammatory cytokines and a significant decrease in blood IL-1β levels in diabetic rats. In support, Li et al. [89] reported a significant suppression of the serum IL-1β level in the BM-MSCs group. This combination is the most potent form of treatment. The activated Th2 cells secrete the anti-inflammatory cytokine IL-13 that suppressing the secretion of several inflammatory cytokines from macrophages and monocytes [90,91]. In the absence of IL-13, the postprandial hepatic glucose metabolism was disrupted, and the glucose uptake in the white adipose tissues was reduced, resulting in insulin resistance, postprandial hyperglycemia, weight gain, decreased oxygen consumption, and increased triglyceride levels in the blood and liver [92]. IL-13 may help mitigate the pro-inflammatory responses and the development of insulin resistance in T2DM [90,93], resulting to a return to normal glucose homeostasis [94]. This study demonstrated the amelioration effect of chrysin, BM-MSCs, and chrysin plus BM-MSCs on IL-13 as an anti-inflammatory in NA/STZ-induced T2DM rats, confirming its anti-inflammatory efficacy. In this context, BM-MSCs and chrysin could reduce the serum concentration and the development of the pro-inflammatory cytokines IL-1β and TNF-α, most likely through the effects of both the compounds in the cellular pathway that improve the metabolism of carbohydrates.

## 5. Conclusions

By lowering inflammation, restoring β-cell functionality, and attenuating insulin resistance whether used alone or in combination, BM-MSCs and chrysin enhanced oral glucose tolerance. The combination of BM-MSCs and chrysin was the most effective treatment. Both chrysin and BM-MSCs may induce insulin sensitization through the downregulation of pro-inflammatory cytokines (such as TNF-α and IL-1β), FFAs, and resistin expression in adipose tissues as well as the upregulation of adipose tissue IR-Bs, IRS-1, and IRS-2, adiponectin expression, and serum IL-13. As a result of their ability to reduce inflammation and improve the glycemic state as well as their involvement in reducing histopathological alterations, we advise combining MSCs and chrysin. Before this combination is authorized for use in the treatment of DM in humans, clinical trials are necessary to evaluate its efficacy and safety.

## Figures and Tables

**Table 1:** Effect of chrysin and BM-MSCs on AUC for OGTT in NA/STZ-induced diabetic rats.

Groups	AUC (mg/dL × 120 min) × 10^2^	% Change
Normal	81.57 ± 4.71 ^a^	
Diabetic control	478.94 ± 21.85 ^d^	487.15
Diabetic treated with chrysin	334.83 ± 28.99 ^b,c^	−30.09
Diabetic treated with BM-MSCs	385.88 ± 10.84 ^c^	−19.43
Diabetic treated with chrysin and BM-MSCs	306.36 ± 30.50 ^b^	−36.03
F-probability	*p* < 0.001	

Data are expressed as mean ± SE. Six samples from each group were detected. Means (with various superscript symbols: a, b, c and d) for each parameter differ significantly at *p* < 0.05. Percentage changes were calculated by comparing the diabetic control with normal and diabetic treated groups with diabetic control. % change = [(Final − Initial)/Initial] × 100.

**Table 2:** Effect of chrysin and BM-MSCs on serum insulin and C-peptide levels in NA/STZ-induced diabetic rats.

Groups	Insulin (ng/mL)	% Change	C-Peptide (pg/mL)	% Change
Normal	2.92 ± 0.10 ^c^		8.93 ± 0.43 ^c^	
Diabetic control	1.03 ± 0.02 ^a^	−64.86	2.53 ± 0.15 ^a^	−71.64
Diabetic treated with chrysin	2.29 ± 0.08 ^b^	122.98	5.1 ± 0.11 ^b^	101.34
Diabetic treated with BM-MSCs	2.43 ± 0.13 ^b^	136.90	6.17 ± 0.27 ^b^	143.70
Diabetic treated with chrysin and BM-MSCs	2.45 ± 0.05 ^b^	138.56	5.83 ± 0.43 ^b^	130.28
F-probability	*p* < 0.001		*p* < 0.001	

Data are expressed as mean ± SE. Six samples from each group were detected. Means (with various superscript symbols: a, b, and c) for each parameter differ significantly at *p* < 0.05. Percentage changes were calculated by comparing the diabetic control with normal and diabetic treated groups with diabetic control. % change = [(Final − Initial)/Initial] × 100.

**Table 3:** Effect of chrysin and BM-MSCs on serum FFAs levels in NA/STZ-induced diabetic rats.

Groups	FFAs (mg/dL)	% Change
Normal	14.72 ± 0.34 ^a^	
Diabetic control	26.45 ± 1.47 ^b^	79.69
Diabetic treated with chrysin	17.56 ± 0.50 ^a^	−33.61
Diabetic treated with BM-MSCs	17.46 ± 0.39 ^a^	−33.99
Diabetic treated with chrysin and BM-MSCs	15.48 ± 0.93 ^a^	−41.47
F-probability	*p* < 0.001	

Data are expressed as mean ± SE. Six samples from each group were detected. Means (with various superscript symbols: a and b) for each parameter differ significantly at *p* < 0.05. Percentage changes were calculated by comparing the diabetic control with normal and diabetic treated groups with diabetic control. % change = [(Final − Initial)/Initial] × 100.

**Table 4:** Effect of chrysin and BM-MSCs on liver glycogen content, G-6-Pase, and glycogen phosphorylase activities in NA/STZ-induced diabetic rats.

Groups	Liver Glycogen (mg/g Tissue)	% Change	G-6-Pase (mg Pi Liberated/g Tissue/Hour)	% Change	Glycogen Phosphorylase (mg Pi Liberated/g Tissue/Hour)	% Change
Normal	36.84 ± 1.10 ^d^		14.89 ± 0.67 ^a^		11.69 ± 0.50 ^a^	
Diabetic control	16.76 ± 0.34 ^a^	−52.69	28.75 ± 0.67 ^d^	93.08	25.78 ± 0.48 ^d^	120.53
Diabetic treated with chrysin	23.54 ± 1.1 ^b^	40.45	18.44 ± 0.73 ^b^	−35.86	18.83 ± 0.29 ^b,c^	−26.96
Diabetic treated with BM-MSCs	21.01 ± 0.25 ^b^	25.36	20.01 ± 0.94 ^b,c^	−30.40	16.47 ± 0.88 ^b^	−36.11
Diabetic treated with chrysin and BM-MSCs	28.19 ± 1.19 ^c^	68.20	21.77 ± 0.63 ^c^	−24.28	19.03 ± 0.56 ^c^	−26.18
F-probability	*p* < 0.001		*p* < 0.001		*p* < 0.001	

Data are expressed as mean ± SE. Six samples from each group were detected. Means (with various superscript symbols: a, b, c, and d) for each parameter differ significantly at *p* < 0.05. Percentage changes were calculated by comparing the diabetic control with normal and diabetic treated groups with diabetic control. % change = [(Final − Initial)/Initial] × 100.

**Table 5:** Effect of chrysin and BM-MSCs on serum IL-1β, TNF-α, and IL-13 levels in NA/STZ-induced diabetic rats.

Groups	IL-1β (pg/mL)	% Change	TNF-α (pg/mL)	% Change	IL-13 (pg/mL)	% Change
Normal	15.23 ± 0.82 ^a^		42.7 ± 1.45 ^a^		116.30 ± 3.01 ^c^	
Diabetic control	91.43 ± 4.55 ^d^	500.33	138.3 ± 7.71 ^d^	223.89	55.13 ± 6.35 ^a^	−52.60
Diabetic treated with chrysin	31.90 ± 2.75 ^b^	−65.11	65.07 ± 1.85 ^b^	−52.95	97.13 ± 1.42 ^b^	76.18
Diabetic treated with BM-MSCs	39.47 ± 5.58 ^b,c^	−56.83	94.5 ± 2.41 ^c^	−31.67	95.67 ± 3.49 ^b^	73.54
Diabetic treated with chrysin and BM-MSCs	49.07 ± 2.56 ^c^	−46.33	44.1 ± 2.08 ^a^	−68.11	93.03 ± 6.36 ^b^	68.75
F-probability	*p* < 0.001		*p* < 0.001		*p* < 0.001	

Data are expressed as mean ± SE. Six samples from each group were detected. Means (with various superscript symbols: a, b, c, and d) for each parameter differ significantly at *p* < 0.05. Percentage changes were calculated by comparing the diabetic control with normal and diabetic treated groups with diabetic control. % change = [(Final − Initial)/Initial] × 100.

**Table 6:** Effect of chrysin and BM-MSCs on integrated density and area fraction levels of immunohistochemical staining of pancreas in NA/STZ-induced diabetic rats.

Groups	Integrated Density	% Change	Area Fraction	% Change
Normal	28,094 ± 237.1 ^e^		13.74 ± 0.12 ^e^	
Diabetic control	772.3 ± 19.0 ^a^	−97.25	0.38 ± 0.01 ^a^	−97.23
Diabetic treated with chrysin	6111 ± 576.2 ^c^	691.27	2.99 ± 0.28 ^c^	686.84
Diabetic treated with BM-MSCs	3917 ± 600.2 ^b^	407.19	1.92 ± 0.29 ^b^	404.74
Diabetic treated with chrysin and BM-MSCs	11,245 ± 196 ^d^	1356.04	5.31 ± 0.19 ^d^	1297.11
F-probability	*p* < 0.001		*p* < 0.001	

Data are expressed as mean ± SE. Four samples from each group were detected. Means (with various superscript symbols: a, b, c, d and e) for each parameter differ significantly at *p* < 0.05. Percentage changes were calculated by comparing the diabetic control with normal and diabetic treated groups with diabetic control. % change = [(Final − Initial)/Initial] × 100.

## Data Availability

All data are contained in the article.
